# Inter-individual variability contrasts with regional homogeneity in the human brain DNA methylome

**DOI:** 10.1093/nar/gku1305

**Published:** 2015-01-08

**Authors:** Robert S. Illingworth, Ulrike Gruenewald-Schneider, Dina De Sousa, Shaun Webb, Cara Merusi, Alastair R. W. Kerr, Keith D. James, Colin Smith, Robert Walker, Robert Andrews, Adrian P. Bird

**Affiliations:** 1 Wellcome Trust Centre for Cell Biology, University of Edinburgh, Edinburgh, Midlothian, EH9 3BF, UK; 2 Wellcome Trust Sanger Institute, Wellcome Trust Genome Campus, Hinxton, Cambridge, CB10 1SA, UK; 3 Neuropathology Unit, Division of Pathology, University of Edinburgh, Edinburgh, EH16 4SB, UK

## Abstract

The possibility that alterations in DNA methylation are mechanistic drivers of development, aging and susceptibility to disease is widely acknowledged, but evidence remains patchy or inconclusive. Of particular interest in this regard is the brain, where it has been reported that DNA methylation impacts on neuronal activity, learning and memory, drug addiction and neurodegeneration. Until recently, however, little was known about the ‘landscape’ of the human brain methylome. Here we assay 1.9 million CpGs in each of 43 brain samples representing different individuals and brain regions. The cerebellum was a consistent outlier compared to all other regions, and showed over 16 000 differentially methylated regions (DMRs). Unexpectedly, the sequence characteristics of hypo- and hypermethylated domains in cerebellum were distinct. In contrast, very few DMRs distinguished regions of the cortex, limbic system and brain stem. Inter-individual DMRs were readily detectable in these regions. These results lead to the surprising conclusion that, with the exception of cerebellum, DNA methylation patterns are more homogeneous between different brain regions from the same individual, than they are for a single brain region between different individuals. This finding suggests that DNA sequence composition, not developmental status, is the principal determinant of the human brain DNA methylome.

## INTRODUCTION

DNA methylation is an epigenetic mark implicated in local adaptation of genome structure to facilitate and stabilize altered activity states. Its importance for brain function is illustrated by the autism spectrum disorders Rett and Fragile X syndrome. Both conditions are primarily caused by conventional mutations that alter the DNA sequence, but each involves downstream aberrations in the DNA methylation system (1,2). Rett syndrome mutations affect the gene encoding methyl-CpG binding protein MeCP2, whereas trinucleotide expansions in the *FMR1* gene attract DNA methylation, which in turn silences the gene. In addition to the evidence provided by these monogenic neurological disorders, it has been shown that DNA methylation is required for normal brain development (3). Moreover, there are reports that DNA methylation impacts diverse brain functions including neuronal activity, learning and memory, drug addiction and neurodegeneration (4–10). The extent to which DNA methylation regulates the gene expression patterns involved in determining cell identity and function in the brain is, however, largely unknown.

Growing interest in the brain DNA methylome requires improved knowledge of its detailed DNA methylation patterns. High coverage nucleotide-resolution DNA methylome data is increasingly available (11,12), but comparison of many samples at this depth is still impractical. To cope with this limitation, methods have been developed that allow analysis of a subset of all genomic sequences (13). Alternatively, affinity purification of methylated DNA using immobilized antibodies against m5C (14) or protein domains from methyl-CpG binding (MBD) proteins (15) is routinely utilized. These methods have the advantage of simplicity, although they tend to focus on regions of the genome where methyl-CpG is clustered. For example, methylated CpG islands (CGIs) and other relatively densely methylated domains are efficiently detected, but CG-poor sequences are underrepresented.

In this study we used MBD-Affinity Purification (MAP) (15) followed by deep sequencing (16) to study DNA methylation patterns in a panel of 43 primary human brain samples from 25 individuals displaying no neurological disorders at the time of death (17). Our study examined eight brain regions altogether, performing comparisons both within and between individual brains. A recent related study using MeDIP followed by deep sequencing reported significant differences in the DNA methylomes from different brain regions and found that inter-individual variations in DNA methylation were small in comparison to variations between tissues (18). We confirm here that cerebellum is an outlier with respect to DNA methylation, but differ from the previous study in seeing very few differences in CG methylation among four cortical regions (occipital, parietal, frontal and temporal lobes), two limbic regions (thalamus and hippocampus) and one brain stem region (pons). This striking homogeneity of DNA methylation patterns was not due to insensitivity of the MAP-seq method, as, unexpectedly, inter-individual variation for each brain region was readily detected and greatly exceeded variation between most brain regions within a single individual.

Our experiments produced several other unanticipated findings. First, the gains and losses of DNA methylation that we observe in cerebellum affect distinct DNA sequence classes: hypomethylation predominantly occurs at CGIs, whereas hypermethylation is largely confined to non-coding DNA. Secondly, a significant fraction of apparent differentially methylated regions (DMRs) on detailed examination turned out to be caused by DNA sequence variation between individuals rather than altered DNA methylation. The persistence of these apparent DMRs despite stringent screening for false positives suggests that genetic variation can be an important potential source of error in DNA methylome analyses. Altogether our findings are compatible with the hypothesis (19–21) that a large fraction of the inter-individual variability of DNA methylomes is due to differences in DNA sequence.

## MATERIALS AND METHODS

### Ethics statement

Work involving the use of human post-mortem brain samples was approved by the Lothian Research Ethical Committee (Ref. 2003/8/37). All samples were anonymized prior to DNA extraction.

### DNA extraction

Post-mortem, histologically graded brain samples were provided by the MRC Sudden Death Brain Bank, Edinburgh (17). Approximately 500–1000 mg of each sample was snap frozen in liquid nitrogen, ground into powder and then incubated in 500 μls of lysis buffer (6 M guanidinium hydrochloride, 30 mM sodium citrate, 0.5% w/v sarkosyl, 0.2 mg/ml proteinase K, 0.2 mg/ml RNase A and 0.3 M β-mercaptoethanol) at 55°C for 4 h. Lysates were extracted once with phenol:chloroform:isoamyl alcohol and once with isoamyl alcohol:chloroform. DNA was then extracted from the aqueous phase by the addition of 1 volume of isopropanol. DNA was re-suspended in 1× TE buffer.

### MBD affinity purification-seq (MAP-seq)

MAP-seq was performed as previously described (15,16,22). Briefly, 70 μgs of fragmented brain genomic DNA were ligated to Solexa paired-end sequencing adaptors. Two independent technical replicates were performed with 35 μgs of DNA, each involving two sequential rounds of chromatography. Polymerase chain reaction (PCR) verified, MAP-purified DNA was then PCR amplified (16 cycles) using primers complementary to the adaptor sequences, following which the DNA was captured on an Illumina flow cell for cluster generation. 37 bp reads were generated using a Genome Analyzer following the standard Illumina protocol. Single-end sequence reads were mapped to the mouse genome (NCBI 36 / hg18) using MAQ (http://maq.sourceforge.net/). Reads with a mapping score greater or equal to 20 were retained (scored based on the BAM tools definition; https://github.com/pezmaster31/bamtools). Individual lanes of sequence were combined as outlined in Supplementary Table S1 prior to downstream analysis. Sequencing data has been deposited in the GEO database (http://www.ncbi.nlm.nih.gov/geo/) under the accession: GSE50960.

### DMR identification strategy

The first step in DMR identification was to define the portion of the genome with robust and interpretable MAP-seq coverage, which we refer to as ‘MAP-regions’. Intervals containing appreciable MAP-seq signal were identified by ‘peak finding’ (parameters for depth, length and gap were four reads, 90 bp and 50 bp respectively; performed using custom scripts). ‘Peaks’ from all 43 brain samples, were merged with those from 8 published MAP-seq data sets for colon, blood and sperm (16). Intervals containing repetitive elements, as defined by repeatmasker, were removed and only ‘MAP-regions’ longer than 250 bp with at least two CpGs were retained (operational minimum for efficient MAP enrichment). Mean read depths were determined for abutting 50 bp windows for each genomic interval for all samples. A nominal read depth offset of 0.1 was applied to prevent infinity values when calculating ratios. Using the ‘limma’ package in R, each sample (expressed as a ratio of the average read depth) was quantile normalized to eliminate read depth variability (All MAP-seq profiles presented represent MAP-seq data normalized in this way). Using the average read depth as a common reference, linear models were generated for each biological variable. These ‘reference’ comparisons were then used to construct secondary models to statistically differentiate between variables of interest (e.g. Male versus Female). Significance levels were subsequently corrected for multiple testing by applying the ‘topTable’ function to the linear model of interest (Benjamini Hochberg method; *P-*value < 0.01). Finally, regions containing at least three significant windows within a 500-bp interval were knitted together as DMRs.

### Bisulfite sequencing

Bisulfite sequencing was performed as previously described (16) with amendments noted in (15) (primers are available on request).

### Genomic annotation

All data was mapped to the (NCBI 36 / hg18) human genome build. Unless otherwise stated, the gene annotation used for all analysis was based on ENSEMBL transcripts (GRCh37 build). For gene location comparisons, DMRs were converted to hg19 and intersections determined based on the following hierarchy: (i) Transcription start site (TSS; ±500 bp), (ii) exonic, (iii) intronic, (iv) upstream of a TSS (+2000 to +501 bp) and (v) intergenic (>2000 bp upstream of TSS). CGIs were defined in a previous study (16).

### Gene expression

#### Analysis of BioGPS gene expression data

Gene expression data was mined from the BioGPS database (http://biogps.org). Expression values were extracted for cerebellum and other brain regions (the median value across temporal lobe, occipital lobe, parietal lobe, hypothalamus, thalamus and pons expression data sets). Gene expression values were then quantile normalized in R and mapped to Ensembl transcripts (hg19). Genes with expression levels in the lowest 25% in both data sets were removed at this point. The significance of differential gene expression associated with DMRs was determined using a Wilcoxen rank sum test.

#### Analysis of Allen brain atlas gene expression data

High spatial resolution human brain gene expression data was extracted from the Allen Brain Atlas database (23,24). Expression data for each brain region (cerebellum, thalamus, hippocampus, pons, occipital, parietal, temporal and frontal) was averaged from all available constituent regions. Differential gene expression was determined on this data without normalization (data was pre-normalized) using the limma package for R. Differentially expressed genes were determined using linear models comparing each brain region to the other regions or pairwise between cortical regions. Cell-type enrichment was determined using expression markers for glia (*GFAP, BIRC5, CD44* and *RIPK1*), purkinje cells (*Calb1, GABRA6, PCP4, PCP2*) and granule cells (*ZIC1, ZIC2, Pax6, Barhl1*).

### Gene ontology (GO) analysis

GO analysis was performed using the R package ‘ChIpPeakAnno’ with multiple testing correction using the Bonferroni method (*P*-value > 0.05).

### Hierarchical clustering

Hierarchical clustering and the associated heatmaps and dendrograms were generated using the ‘hclust’ function in R. Clustering on methylation levels was performed on the mean MAP-seq coverage for ‘MAP-regions’ or for individual genomic features as described in the text. For gene expression, clustering was performed on combined data from the Allen Brain Atlas (see **Gene Expression** for details).

### Principle component analysis (PCA)

PCA was performed on the mean MAP-seq values for all ‘MAP-regions’. Prior to PCA a lower read depth limit of 0.1 was applied and sex chromosomes were removed. PCA was performed using the ‘prcomp’ function and the results were plotted using the ‘plot3d’ package in R.

### Enhanced reduced representation bisulfite sequence (eRRBS) analysis

Published eRRBS data for neuronal and non-neuronal cortical cell fractions was extracted from the GEO repository (GSE50852) (25). CpG sites with a minimum coverage of 10 reads were retained and methylation values for neuronal and non-neuronal fractions averaged across the biological replicates and DNA strands. Brain region DMR locations were converted to hg19 and then intersected with the averaged eRRBS data. DMRs were plotted only if they intersected data from both neuronal and non-neuronal data sets. Note that 5535 (∼78%) hypomethylated and 2119 (∼23%) hypermethylated DMRs were analysed and statistical assessment was performed using a Wilcoxen rank sum test.

### Melt curve analysis

To determine the presence of polymorphic sequence, regions spanning in DMRs were amplified using quantitative PCR on a LightCycler 480 using the SYBR Green I Master mix (Roche). DNA melting properties were determined by a melt curve cycle and the data visualized as plots of temperature versus the ratio of delta fluorescence/delta temperature (-d/dT).

## RESULTS

### Differential methylation in primary brain samples

**Figure 1.**
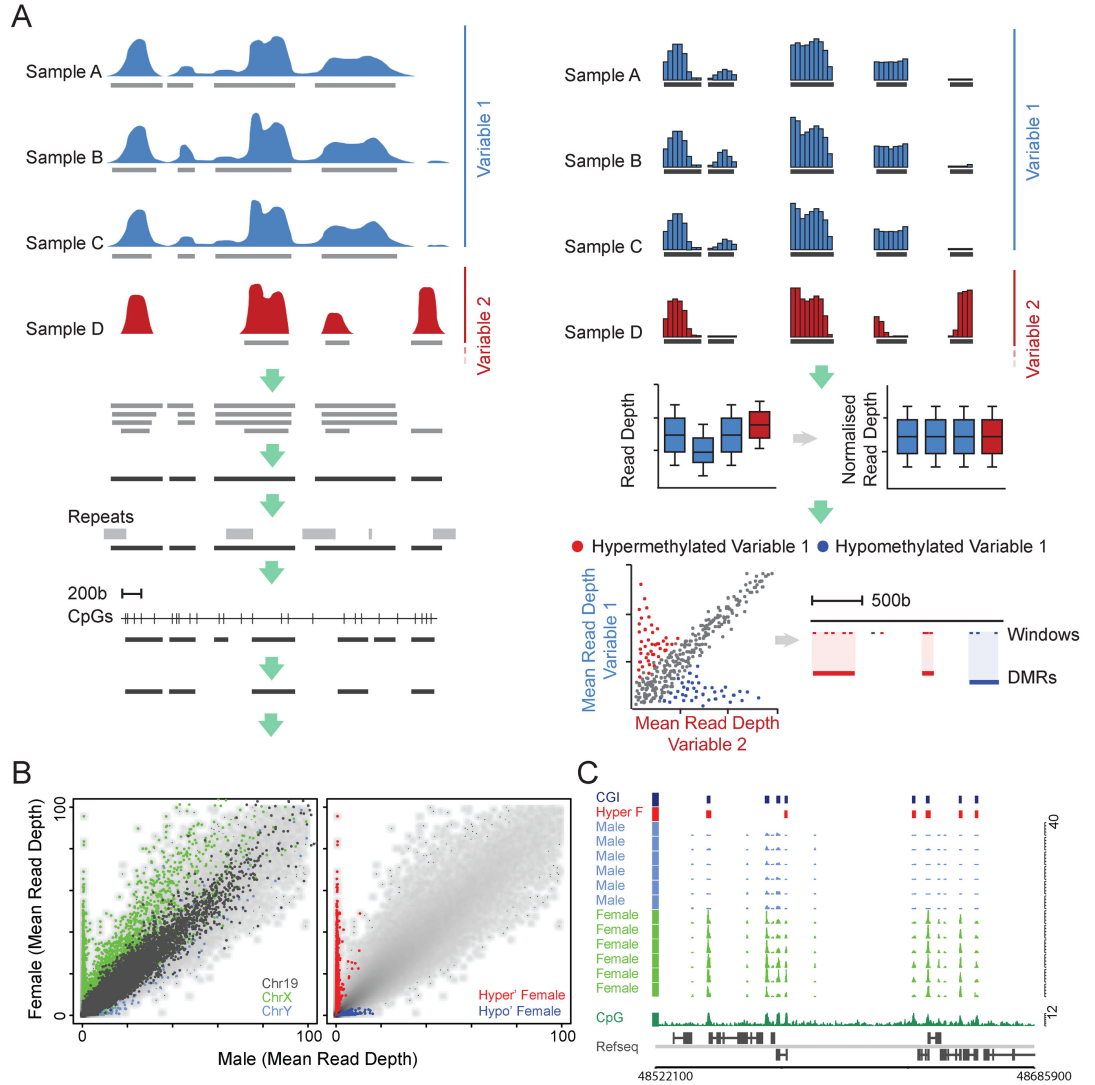
DMR identification strategy. (A) Schematic representation of the DMR identification procedure. Indicated is a hypothetical data set where samples representing two biological variables (red and blue) are processed to identify regions of significantly differential methylation (see text for details). (B) Scatterplots depicting male and female averaged MAP-seq read depths for all MAP regions. The first plot shows the relative read depths for all windows on chromosomes X (green), Y (pink) and 19 (grey; left panel). The second plot shows the same data as the first but highlights windows which were identified using the MAP-seq analysis strategy as being significantly hyper- or hypomethylated in female versus male samples (red and blue, respectively). (C) A representative region of the X chromosome (chrX: 48513000–48740500; hg18) showing the MAP-seq profiles for male and female samples (pink and green, respectively). Female-specific hyper- and hypomethylated DMRs are indicated (red and blue boxes, respectively). CpG islands (CGIs; dark blue boxes), forward and reverse strand refseq genes (upper and lower panel, respectively) and CpG density profiles (CpGs per 100 bp; dark green plot) are displayed for reference.

Methylated DNA from each brain sample was isolated, subjected to deep sequencing in duplicate and the resulting data was combined for further analysis (Supplementary Table S1). To identify regions of differential methylation associated with defined biological variables, we developed a custom computational pipeline to process MAP-seq data. First, regions were identified by low stringency ‘peak finding’ for each brain sample and these were merged with regions of enrichment identified previously in colon, blood and sperm (16). In total 373 169 regions spanning 138 786 961 bp (4.51% of the haploid genome) were identified. Repeat sequences were removed and only intervals longer than 250 bp with at least two CpGs were retained. The filtering resulted in the selection of 97 864 MAP regions with slightly elevated G+C composition and CpG density compared to the genome average. Though conservative, this process allowed robust detection of the methylation status of 1.9 million (13.9%) of all non-repetitive CpGs in the human genome. Constitutively hypomethylated CpG islands (CGIs) were excluded, as they are not retained by the MAP procedure. Mean read depths were calculated for abutting 50 bp windows across all MAP regions, for each data set. Following this spatial rationalization, sequence depth variability was eliminated using quantile normalization. Linear modelling was applied to collapse the data to the biological variable of interest allowing identification of differentially methylated windows. Each window was then corrected for multiple testing using the Benjamini–Hochberg method (*P-*value < 0.01). Finally, regions containing more than two significant windows within a 500-bp interval were knitted together to define a robust set of DMRs, which were then used for downstream analysis. The analysis is summarized in Figure 1A and described in detail in Materials and Methods.

To validate the procedure, we attempted to identify regions of differential methylation that are associated with female X-inactivation across all 43 brain samples (Figure 1B and C) (26). Scatter plots representing the sample-averaged read depth for males (*n* = 36) and females (*n* = 7) showed clear enrichment of regions on the X chromosome in female and a reciprocal enrichment of Y-linked windows in male (Figure 1B). In contrast, no differential enrichment was observed for autosomal chromosome 19, as expected (Figure 1B). To quantify these empirical observations, we identified all windows presenting differential methylation levels (*P*-value < 0.01) and found 766 hypermethylated DMRs, of which 760 proved to be X-linked. In male samples, 33 hypermethylated DMRs were detected of which 29 were Y-linked. Robust detection of known DMRs associated with X-chromosome inactivation confirmed the efficacy of this analysis strategy when applied to MAP-seq data.

### Methylation levels distinguishes cerebellum from other brain regions

**Figure 2.**
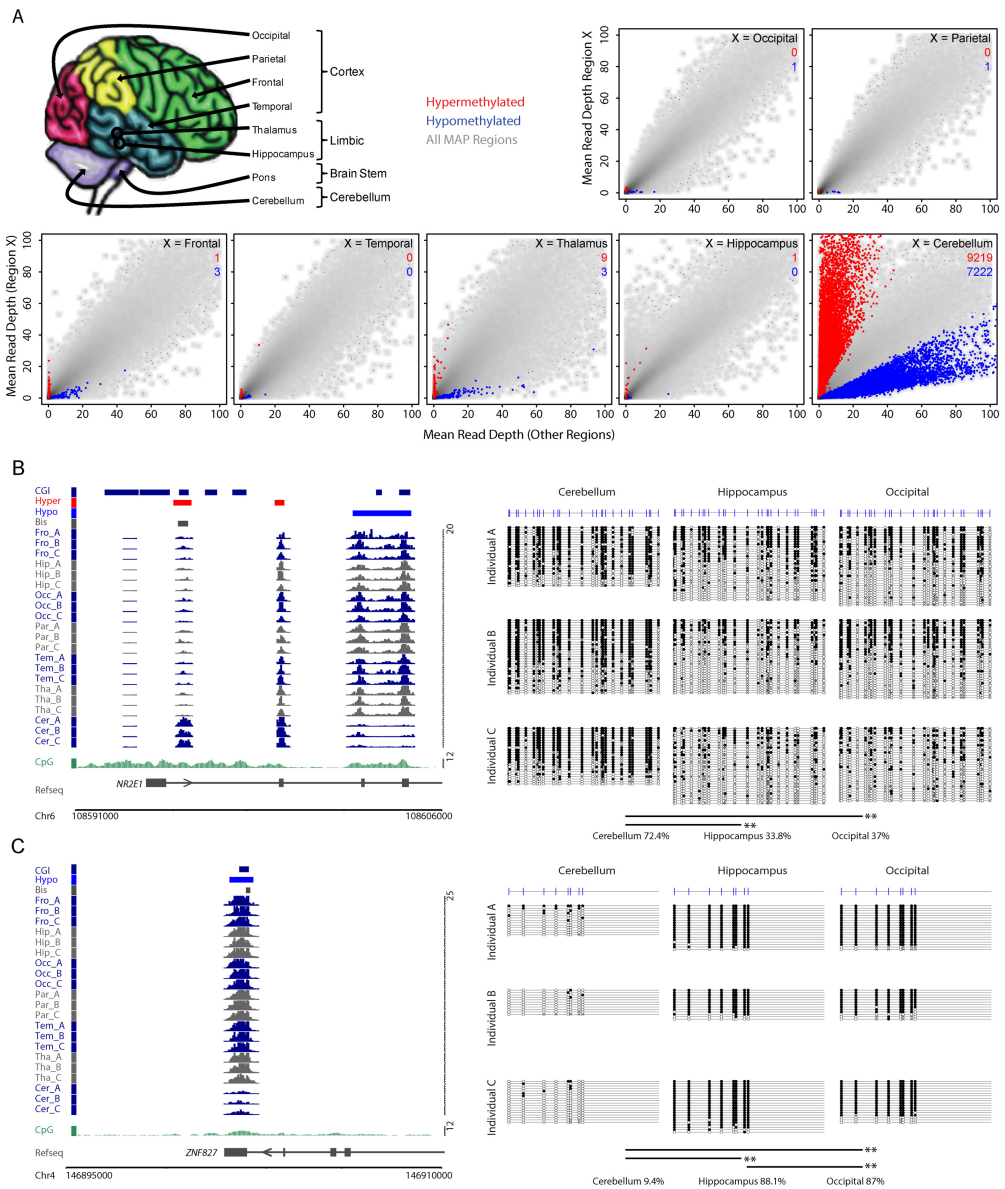
The methylome of cerebellum is distinct within the human brain. (A) Schematic representation of the eight brain regions investigated followed by scatterplots depicting the mean read depths for all 50 bp windows for each of the brain region versus the mean of the other brain regions. Region-specific hyper- and hypomethylated windows are indicated for each brain region (red and blue points, respectively). The number of hypermethylated (red) and hypomethylated (blue) DMRs merged from the highlighted windows are indicated for each comparison. (B) The left panel depicts the MAP-seq profiles for each brain region (dark blue and grey) at the *NR2E1* locus (chr6 108590316–108605633; hg18 genome build). Hyper- and hypomethylated cerebellum DMRs and the location of the bisulfite amplicon are indicated (red, blue and grey boxes, respectively). CpG islands (CGIs; dark blue boxes), forward and reverse strand refseq genes (upper and lower panel, respectively) and CpG density profiles (frequency per 100 bp; dark green plot) are displayed for reference. The right panel shows the bisulfite sequencing result confirming that cerebellum is more heavily methylated relative to occipital and hippocampal samples in all three individuals as predicted by the MAP-seq analysis. Open and filled circles represent non-methylated and methylated CpG sites, respectively. Each line depicts a single sequenced PCR product and the CpG positions are indicated by vertical strokes. The significance of differential methylation distribution between samples at the DNA strand level was determined using a Wilcoxen rank sum test, and *P-*values are indicated as either <0.05* or <0.01**. (C) The left panel depicts the MAP-seq profiles for each brain region at the *ZNF827* locus (chr4 146894705–146909705; hg18 genome build). The right panel shows the bisulfite sequencing result confirming that cerebellum is hypomethylated relative to occipital and hippocampal samples in all three individuals as predicted by the MAP-seq analysis. The colouring and layout is presented as for panel (B).

We used this verified data processing strategy to determine the DNA methylation patterns in eight brain regions of three independent clinically normal individuals. The material included four regions of the cerebral cortex (occipital, temporal, parietal and frontal lobes), two regions of the limbic system (thalamus and hippocampus), the brain stem (pons) and the cerebellum (Figure 2A). Each brain region was compared to the mean MAP-seq read depth from the other samples (pons was excluded from this analysis due to insufficient sequence depth). Scatter plots of each comparison with differentially methylated windows in red and blue representing hyper- and hypomethylated, respectively, are presented in Figure 2A. There was remarkably little inter-region variability with the marked exception of cerebellum, which, as reported previously (18), behaved as a notable outlier with respect to all other samples tested (Figure 2A). Altogether cerebellum had 16 441 DMRs of which 9219 were hypermethylated and 7222 were hypomethylated relative to the other brain regions (Figure 2A). Given the magnitude of this difference and the relative similarity of the other samples, we were concerned that by focussing on small brain regions we might have missed DMRs that potentially differ between higher order brain compartments. To test this possibility we repeated our analysis with the samples grouped according to their cortical, limbic or cerebellar domain of origin (Supplementary Figure S1A–C). Marginally higher levels of differential methylation were detected among these higher order brain compartments, but these remained much less than that between all these regions and cerebellum (Supplementary Figure S1A–C).

To validate our results we performed bisulfite sequencing for four candidate DMRs (three intragenic and one intergenic) in cerebellar, hippocampal and occipital samples (Figure 2B and C and Supplementary Figure S2A and B). The results confirmed the MAP-seq analysis, as all samples except cerebellum showed closely similar DNA methylation profiles. For example, the *NR2E1* gene, which encodes the TLX nuclear receptor, is marked by a conspicuous hypermethylated DMR within the first intron in cerebellum, whereas the same region is hypomethylated in the occipital lobe and hippocampus (Figure 2B).

### Hypo- and hypermethylation at cerebellar DMRs affects distinct sequence categories

**Figure 3.**
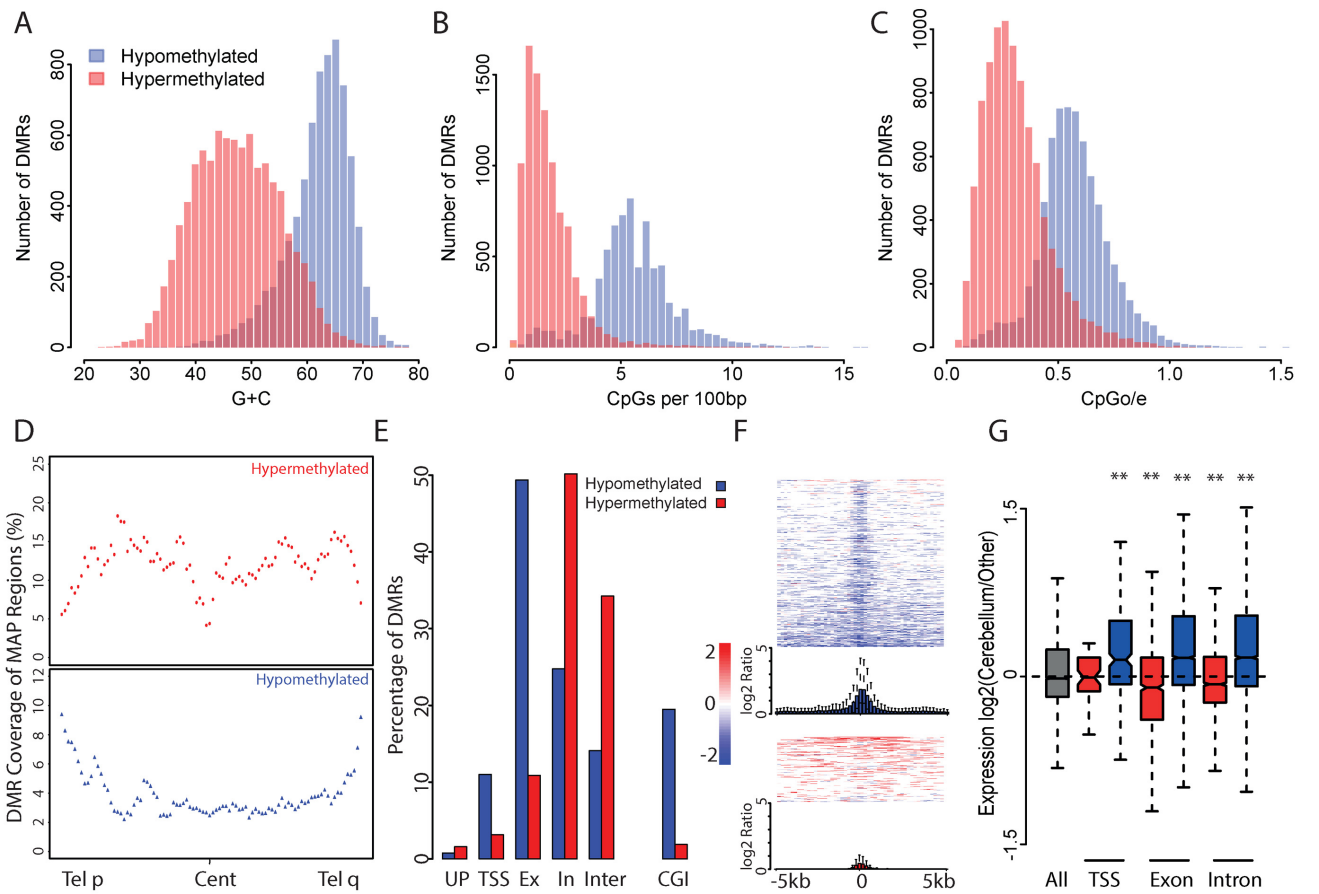
Hyper- and hypomethylated cerebellum DMRs show distinct characteristics. (A–C) Hypomethylated cerebellum DMRs show a base composition typical of CGIs, whereas hypermethylated DMRs more closely resemble the physical properties of the bulk genome. Histograms show the distribution of G+C composition (A), CpG density (CpGs per 100 bp; B) and CpG observed/expected ratio (CpGo/e; C) for hypo- and hypermethylated DMRs (blue and red, respectively). (D) Plots representing the distribution of cerebellum DMRs across an average autosome show that hypomethylated DMRs (blue; lower panel) preferentially localize towards the chromosome ends, whereas hypermethylated DMRs (red; upper panel) show a reciprocal distribution. Coverage is measured as the number of DMRs relative to that of MAP-regions in each percentile window across all autosomal arms. (E) Bar plots show that hypomethylated DMRs (blue) preferentially localize to CGIs, exons and TSSs, whereas hypermethylated DMRs (red) are preferentially localized to intronic and intergenic sequences (see text for details). (F) Heatmaps, which span 10 kb centred on a CGI, illustrate that hypomethylated DMRs co-localize with CGIs, whereas hypermethylated DMRs are more randomly distributed. Plotted data represent the log2 ratio of methylation level in cerebellum versus the other brain regions. Summary boxplots for hypomethylated (blue) and hypermethylated DMRs (red) depict the log2 differential methylation level. (G) Expression levels of DMR-associated genes show a significant anti-correlation with DMR methylation status irrespective of DMR location. Boxplots show the expression levels of genes which have DMRs located at their TSS, exons or introns (significance of each comparison is displayed as for Figure 2B). Genes were categorized as overlapping a hyper- or hypomethylated cerebellum DMR (red and blue, respectively).

To better understand the functional implications of cerebellum-specific DNA methylation patterns we characterized the DNA sequences within DMRs. Surprisingly, hypo- and hypermethylated cerebellar DMRs are derived from distinct DNA sequence categories. Hypomethylated DMRs consistently have a high G+C composition, high CpG observed/expected (o/e) and high CpG density, suggesting that most are derived from CGIs. Hypermethylated DMRs, on the other hand, resemble bulk genomic DNA in each of these properties, being AT-rich and CpG-deficient (Figure 3A–C and Supplementary Table S2). Further evidence that the DNA sequences affected by loss or gain of methylation are distinct came from an analysis of their chromosomal locations. Hypomethylated DMRs are substantially enriched towards chromosome ends, whereas hypermethylated DMRs showed a reciprocal pattern of enrichment (Figure 3D and Supplementary Figure S3A).

We next asked if this unanticipated distinction between the DMR sets was reflected in their locations with respect to genes. Locations were classified as upstream of a TSS (−2000 to −501 bp), overlapping a TSS (±500 bp), exonic, intronic or intergenic (Figure 3E). Consistent with their DNA sequence properties, hypomethylated DMRs preferentially co-localize with CGIs. These mostly occur within the gene body, particularly over exons and, to a lesser extent, TSSs (Figure 3E). Conversely, hypermethylated DMRs are preferentially found outside TSSs and exons, being instead enriched between genes and in introns (Figure 3E). In agreement with these findings, hypomethylated DMRs within a 5-kb window surrounding a CGI tend to centre on the CGI, whereas hypermethylated DMRs show a random distribution (Figure 3F). We conclude that the cerebellar DNA methylome differs from other brain regions tested in this study in two complementary ways: (i) methylation at specific CGIs, particularly those found at exons (16), is reduced; (ii) methylation at sites between genes or in introns is increased.

As DNA methylation is associated with transcriptional repression, we looked for a correlation between DMRs and gene expression. Gene expression data was extracted from the BioGPS database and cerebellum was compared with the median expression value of appropriate data sets for other brain regions (temporal lobe, occipital lobe, parietal lobe, hypothalamus, thalamus and pons). Once normalized, the expression of genes overlapping cerebellum-hypermethylated and cerebellum-hypomethylated DMRs were compared (Supplementary Table S3). Consistent with published observations in other systems (27–29), we found that expression was anti-correlated with the DNA methylation level of the associated DMR (Figure 3G). Cerebellum-specific hypomethylated DMRs consistently showed a stronger correlation between gene expression and hypomethylation compared with other brain regions. This remained true even when DMRs were separated according to their location with respect to genes, suggesting that the correlation is not exclusively attributable to hypomethylation at the TSS (Figure 3G and Supplementary Table S3). We identified ∼1000 transcripts that possess hypo- and hypermethylated DMRs simultaneously. This category showed no significant expression difference between cerebellum and the other brain regions (data not shown).

We next asked whether DMR-associated genes that show differential expression are involved in specific biological processes. Genes overlapping DMRs that are hyper- or hypomethylated in cerebellum were found to be enriched for ontology terms associated with neuronal development and function (21 of 42 and 14 of 22 for hyper- and hypomethylated, respectively; Supplementary Tables S4 and S5). In addition, there is evident enrichment for processes involved in cell morphology, which are likely to be involved in neuronal function. Numerous genes implicated in development and differentiation also emerged from the gene ontologies, consistent with previous studies showing that developmentally important genes functions often show specific DNA methylation changes (15).

### Hierarchical clustering identifies individual DNA methylation fingerprints

**Figure 4.**
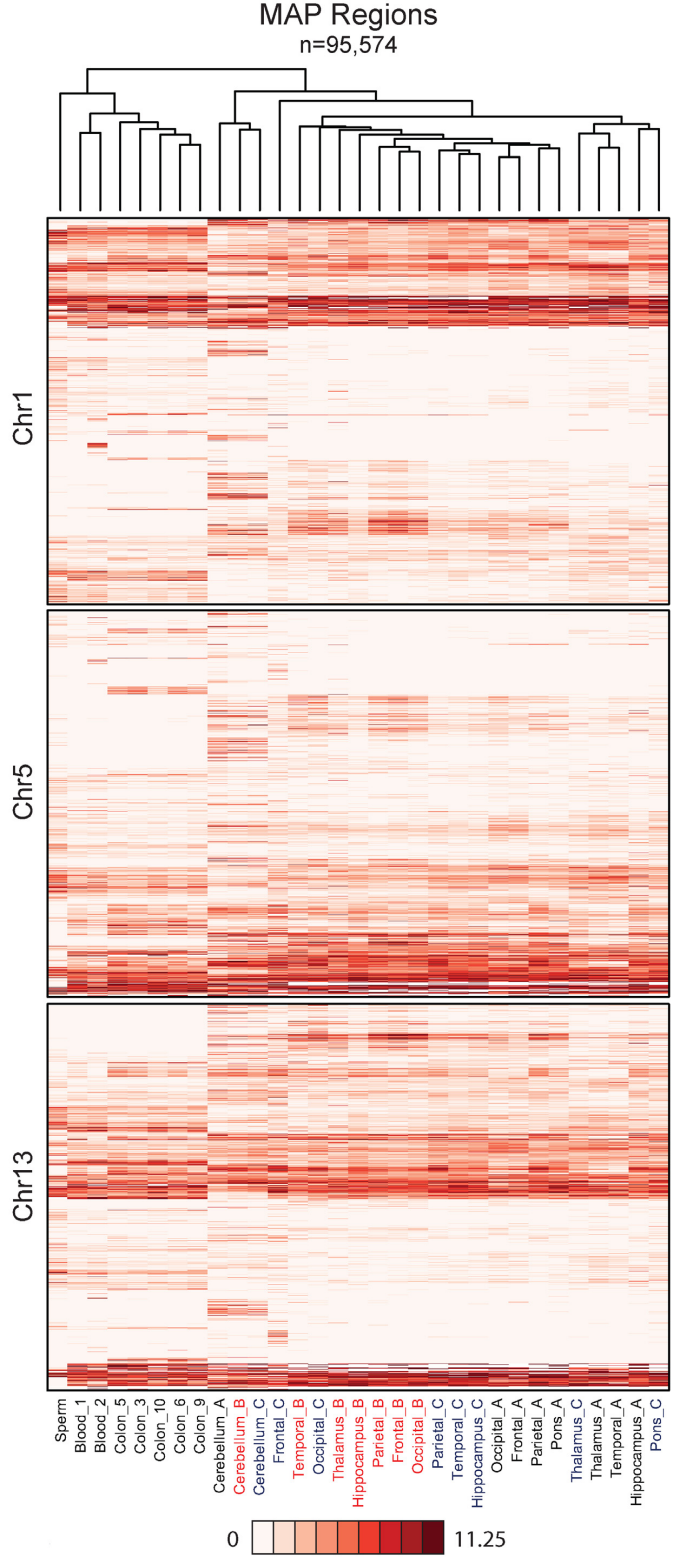
Hierarchical clustering identifies a high level of concordance between the methylomes of different brain regions from the same individual. Heatmaps show the average read depth for all ‘MAP-regions’ on chromosomes 1, 5 and 13. Columns are ordered based on clustering for all autosomes as illustrated by the dendrogram.

The contrast between the DNA methylome of cerebellum and that of all the remaining tested regions of the brain raised the possibility that these prominent differences were obscuring more subtle patterns in our data set. To expose hidden variation, we performed unsupervised clustering of the samples, with the addition of colon, blood and sperm data (16) as references to aid interpretation. For all autosomal MAP regions (*n* = 95 574) we found a clear distinction between the brain, blood, colon and sperm, which agrees with their distinct developmental origins (ectoderm, mesoderm, endoderm and germ line, respectively; Figure 4). Consistent with our previous results, cerebellum segregates into a discrete cluster with respect to all other brain regions, but there is little if any clustering of other brain regions with respect to functional relatedness. Instead, the samples preferentially cluster according to the individual from which they were derived (denoted by A, B and C; Figure 4). These observations were further substantiated using PCA performed on the same data (Supplementary Figure S4A). Repeating the unsupervised clustering analysis to look specifically at CGIs (Supplementary Figure S4B–D) and other discrete regions of the genome (data not shown) again showed segregation based on the individual of origin rather than the brain region. These findings demonstrate that the failure to find inter-regional differences in brain methylomes is not due to the insensitivity of the MAP-seq method. The surprising conclusion is that, for brain regions other than cerebellum, DNA methylation patterns are more similar to other brain regions from the same individual, than they are to the equivalent brain region from a different individual.

The similarity of DNA methylomes in non-cerebellar brain regions might in theory be caused by differences in cellular composition. For example, a high proportion of glia relative to neurons in these samples might allow glial gene expression patterns to mask differences in neuronal gene expression patterns between the regions. To assess this possibility we compared the different brain regions with respect to expression of glial-specific genes using published data (23,24). The results showed that the proportion of glial-specific gene expression is relatively constant in all tested brain regions and is therefore highly unlikely to explain the constancy of brain methylome patterns (Supplementary Figure S5A). As a control for the reliability of this analysis, mRNAs characteristic of purkinje cells and granule cells were greatly enriched in the cerebellum as expected (Supplementary Figure S5A).

Given that marker gene expression is an inherently fallible method to stratify cell types, we wished to further investigate cellular composition using an alternative and more directly comparable data set. For this we overlaid our DMR sets against eRRBS data generated from neuronal and non-neuronal cortical cell fractions (25). Strikingly, no quantitative difference was observed between these fractions for either hyper- or hypomethylated DMRs, which supports our contention that distinctive cerebellar methylation characteristics do not arise from a shift in abundance of neuronal to non-neuronal cell types (Supplementary Figure S5B).

While DNA methylomes are relatively invariant between non-cerebellar brain regions, patterns of gene activity differ greatly. Analysis of published data (23,24) shows that thousands of genes are differentially expressed between human brain regions comprising the limbic system, cortical regions and brain stem (Supplementary Figure S6A). Pairwise comparison of transcription in the four cortical regions showed, that while being relatively homogeneous, they still differ by more than 200 differentially expressed genes. It follows that that major differences in gene expression occur against the background of a relatively constant epigenome (Supplementary Figure S6A). Cluster analysis confirms that cerebellum is the most distinct brain substructure with respect to gene expression (Supplementary Figure S6B). Given differential gene expression, we expected that these variations would cluster by brain region between individuals, whereas we again observed preferential clustering between different brain regions of a single individual, particularly within cortical regions. This surprising finding suggests that gene expression, as for our methylation analysis, varies more within a single brain region between individuals than between other regions of the brain (Figure 4 and Supplementary Figure S6A).

### Individual-specific DNA methylation patterns

**Figure 5.**
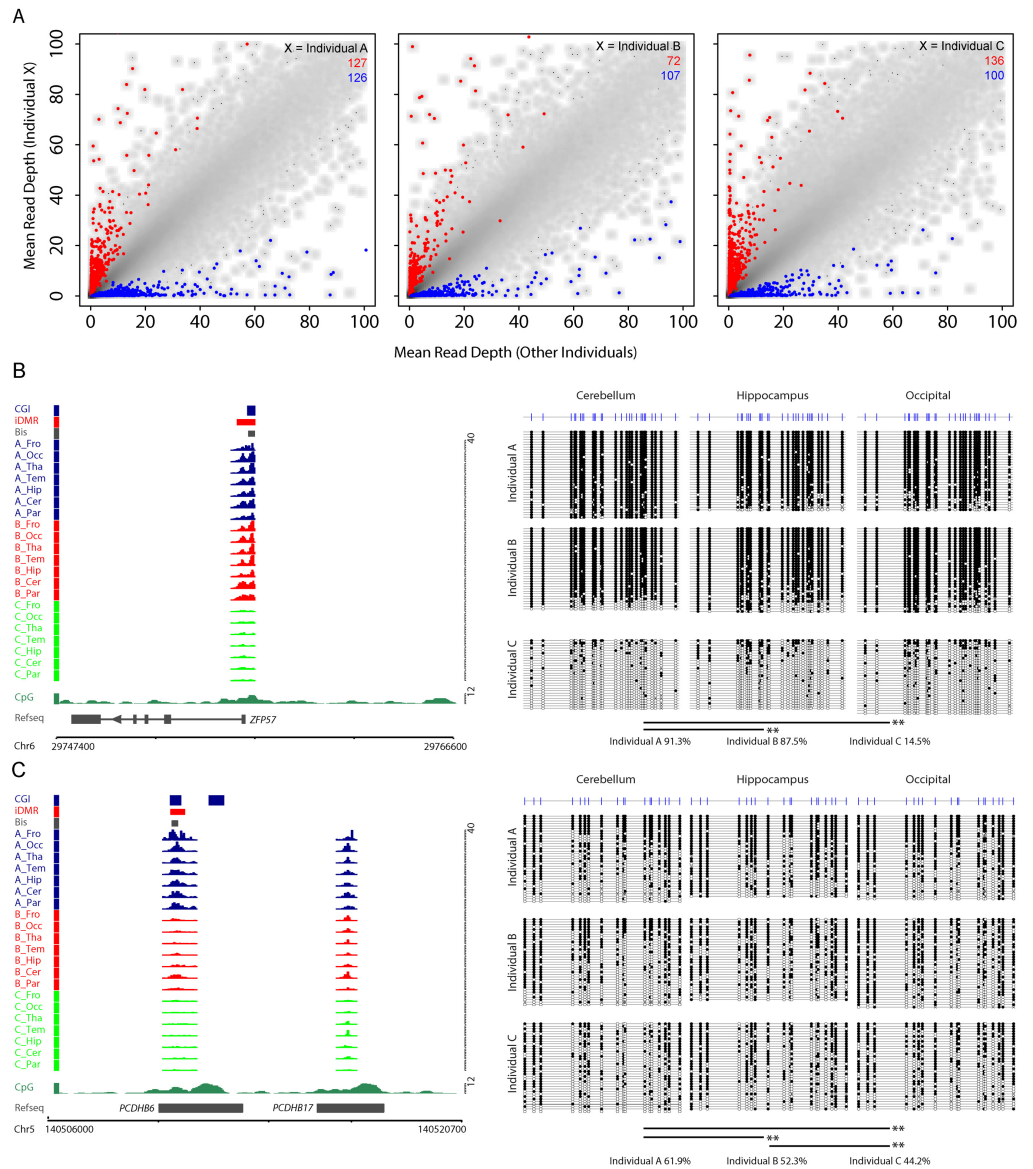
Identification of individual-specific DMRs (inDMRs). (A) Scatterplots depicting the averaged read depths for each individual versus the mean of the other two individuals. Individual-specific hyper- and hypomethylated DMRs are indicated for each individual (red and blue points, respectively). The number of hypermethylated (red) and hypomethylated (blue) DMRs are indicated for each comparison. (B) The left panel depicts the MAP-seq profiles for each individual (Individual A, darkblue; Individual B, red; Individual C, orange) at the *ZFP57* locus (chr6 29746600–29766600; hg18 genome build). The right panel shows the bisulfite sequencing result confirming that Individual C has reduced methylation levels relative to Individuals A and B as predicted by the MAP-seq analysis. (C) The left panel depicts the MAP-seq profiles for each individual (Individual A, darkblue; Individual B, red; Individual C, orange) at the *PCDHB6* locus (chr5 140505700–140520700; hg18 genome build). The right panel shows the bisulfite sequencing result confirming that the methylation level is the highest in Individual A and lowest in C as predicted by the MAP-seq analysis. The layout for (B) and (C) are presented as for Figure 2B.

To investigate further inter-individual variation between DNA methylation patterns, we grouped the brain regions by individual and extracted DMRs (inDMRs) showing consistent enrichment or depletion of MAP-seq signal. As anticipated, specific methylation ‘fingerprints’ emerged for each individual (Figure 5A). In total, 421 inDMRs were identified as hypo- or hypermethylated in at least one of the three individuals (Figure 5A and Supplementary Table S6). We attempted to validate four candidate inDMRs for each of the three individuals by bisulfite sequencing cerebellum, occipital and hippocampal samples (Figure 5B and C and Supplementary Figures S7 and S8). Three of these regions were confirmed, but one apparent inDMR located on chromosome 6 could be amplified from individuals A and B but not from individual C (Supplementary Figure S8B). DNA sequencing revealed an intergenic insertion of a CpG-rich Alu element in individual C, which we confirmed to be heavily methylated (Supplementary Figure S8C and D). It is conceivable that hypermethylation of the element has a functional impact, but the lack of overlap with a known gene makes this possibility difficult to assess. Importantly, the primary change detected in this case is genetic, leading to a secondary alteration in the local epigenetic environment.

To determine if inDMRs manifest in individuals A-C show hyper-variable methylation levels among unrelated individuals, we analysed MAP-seq data for a further 20 hippocampal samples (individuals D–W). Normalized MAP-seq signal for all MAP-regions across the hippocampal sample was compared to the mean signal for all individuals and expressed as log2 ratios for each individual. Using the standard deviation of these ratios as a metric of methylation variance, methylation levels at MAP-regions associated with the iDMRs were found to vary significantly across all 23 unrelated hippocampal samples (Supplementary Figure S9; Wilcoxen rank sum test statistics relate to the comparison of all inDMRs versus the invariant MAP-regions). In addition to the known inDMRs, other MAP-regions showed elevated variance suggesting that these too harbour inDMRs, which vary in individuals D–W (data not shown). Further validation would be required to test the credentials of these putative inDMRs.

During our efforts to identify sites of differential methylation it became apparent that genetic variability between human samples can give rise to false positives. While this is internally controlled when comparing samples from a single individual, there is a clear risk when comparing between individuals that are not genetically identical. The problem is illustrated in Supplementary Figure S8 where an individual-specific MAP-seq signal could be directly attributed to the insertion of a CpG-rich Alu element within an otherwise CpG-deficient locus (individual C). In another instance where MAP-seq detected a potential DMR but no differential methylation could be measured by bisulfite sequencing (Supplementary Figure S10A and data not shown), the region was found to contain seven putative single nucleotide polymorphism sites (SNPs; identified using data from the HapMap project; (30)). PCR amplification of this locus confirmed the correct fragment length but gave individual-specific products in terms of DNA melting properties (Supplementary Figure S10B). Sanger sequencing confirmed the existence of two polymorphic sites in the three individuals both resulting in CpG to CpA transitions (Supplementary Figure S10B). It is tempting to postulate that this resulted in reduced affinity for the MBD column due to loss of CpGs. Closer inspection revealed, however, that the presence of two SNPs (Chr8 1133198 and 1133226) prevented genomic alignment. In light of this observation we performed melt-curve profiling on a further nine inDMRs. Of the regions tested, we found that one-third (3 of 9) contained polymorphisms (Supplementary Figure S10C). While most SNPs are tolerated during alignment, multiple proximal SNPs or more extensive insertions or deletions will disrupt correct mapping and register artefactually as DMRs. Through our conservative filtering strategy, which eliminates repeats and low CpG density sequences, we anticipate that technical false positives of this kind represent a minority of DMRs that we detected.

## DISCUSSION

### The human brain DNA methylome and development

The possibility that alterations in the DNA methylome, triggered by the environment, infection or the passage of time, contribute to phenotypes, such as aging and susceptibility to disease, is widely acknowledged. Of particular interest is the human brain, which can acquire long-term conditions, such as schizophrenia, bipolar disorder, autism, drug addiction, etc. The molecular basis of these conditions is largely uncertain, leaving open the possibility that epigenetic mechanisms play a role. The technical difficulty of sampling human brain has limited most large-scale studies to blood DNA, but the availability of human brain banks permits comparative global analysis of the brain methylome. A previous study using MeDIP-seq detected major differences between cerebellum and other regions (18), an observation that is confirmed here. In that study, regions of the cerebral cortex, however, were found to differ significantly and showed methylomes that were more different from each other than were methylomes of the same brain region between individuals. This is the reverse of our findings, which detected few regional differences except in cerebellum, but significant inter-individual variability. The discrepancy may be due to the different brain regions that were chosen in the two studies, or it may be due to the different technologies (MeDIP versus MAP) used to analyse DNA methylation. Another difference between the previous and present analyses is the selection strategy for DMRs. The former applies a variance metric followed by selection of the top ranked scores for downstream analysis, whereas here a stringent linear modelling strategy that only identifies highly reproducible DMRs is applied (18). This technical distinction may account for the differences observed between the two studies.

The role of changing patterns of DNA methylation in mammalian development has been demonstrated by its involvement in genomic imprinting, X chromosome inactivation and the silencing of germ-line specific genes in somatic cells. These phenomena share the characteristic that they arise early in development and affect a broad range of tissues and cell types. Less clear is the role of DNA methylation in regulating gene expression during the terminal stages of differentiation within an organ. Analysis of *in vitro* differentiating neurons indicated that, while some changes occur early, relatively few DNA methylation changes at GC-rich regions accompany terminal differentiation (31). This was confirmed in cells derived from the *in vivo* immune system of mice, where the methylome remained largely constant despite major changes in gene expression between various immune cell types (28). The findings presented here partially agree with these precedents, as, with the exception of cerebellum, different brain regions have indistinguishable methylomes despite many thousands of gene expression differences between them. Might this unexpected homogeneity be due to the insensitivity of MAP-seq? This is unlikely, as methylome differences were easily detected between individuals, as discussed below. Another hypothetical possibility is that the MAP-seq signal from neurons is swamped by that of a more abundant cell type, such as glia, thereby masking significant differences at the neuronal level. We investigated this and found that the expression of a glial signature gene set was similar between all of the brain regions tested, including cerebellum, even though the latter showed large-scale methylome differences compared to all the other regions. If the proportion of glia were to be much higher in non-cerebellar regions, we would expect higher expression of signature glial genes, which was not observed. In addition, comparison with a published eRRBS data set identified no quantitative difference in methylation levels between neuronal and non-neuronal cell fractions at cerebellum DMRs (25). The data are compatible with the view that dynamic changes in the DNA methylome play a minor role in terminal differentiation of most regions of the brain, including brain stem, cortex and limbic regions.

In contrast to the uniformity of most brain methylomes, the cerebellum displayed a large number of DMRs. The cerebellum is unusual in that it is composed of a very high proportion of neurons, the majority of which are granule cells (∼10^10^ cells). By combining MAP-seq data with gene expression data, a strong inverse correlation was observed between relative gene expression and levels of DNA methylation. Whether this correlation reflects a causative relationship remains unknown, but the fact that many DNA methylation differences are concentrated in gene bodies, suggests that promoter methylation status is probably not a major driver of these differences.

### Inter-individual methylome variation—genetic or epigenetic?

While non-cerebellar regions gave uniform methylomes within an individual, we found abundant evidence for variation between individuals. In fact the individual differences in, for example, the hippocampus methylome, were much greater than those between hippocampus, cortex and thalamus in a single brain. Interestingly, a parallel finding emerged from gene expression studies (Supplementary Figure S6). Once again there was a tendency for different brain regions, particularly the cortices, from one individual to be more similar in gene expression than was any single brain region when compared between different individuals. This is a surprising result, given that the characteristics of a particular tissue are thought to be a reflection of its constituent gene expression patterns. The data suggest that the average cell-type composition of different brain regions (other than cerebellum) is similar.

The DNA methylome and gene expression differences between individuals may arise in either of two ways: (i) through purely epigenetic differences between them that arose during development or in response to environmental impacts; (ii) as a secondary consequence of genetic differences. Analysis of allele-specific differences in DNA methylation has shown that they often coincide with heritable genetic differences (19–21,32). The implication is that much epigenetic variability is secondary consequence of genetic variation. Indeed, detailed analysis of DNA methylation patterns has shown that the primary determinant is genetic, mediated in particular by the effects of DNA sequence on the interaction with DNA binding proteins (11). In the present study we found in a minority of cases that what appeared to be epigenetic variation by MAP-seq was in fact caused by genetic changes at the site in question. In these cases the assay method was unable to distinguish genetic from epigenetic variation resulting in false-positive DMRs. More subtly, it remains possible that local or remote DNA sequence variation underlies much of the DNA methylation differences that we observe. This could explain why different regions of the brain in one individual have uniform methylomes, whereas methylomes of a single brain region vary between individuals. Despite their diverse developmental origins, the brain regions tested here evidently share the same DNA sequence and this may be the decisive factor in setting up the epigenome. Individuals, on the other hand, are genetically distinct and this could explain why the epigenome of, for example, the thalamus in one individual differs from epigenome of the thalamus in a different brain. Our data suggest that DNA methylation is unexpectedly constant between brain regions and fits with the notion that many differences in DNA methylation in the brain are determined genetically.

## SUPPLEMENTARY DATA

Supplementary Data are available at NAR Online.

SUPPLEMENTARY DATA
