# Supplementary material for: Inter-individual variability contrasts with regional homogeneity in the human brain DNA methylome
Source: Nucleic Acids Res. 2015 Jan 8;43(2):732–44. doi: 10.1093/nar/gku1305 (PMC4333374; doi:10.1093/nar/gku1305)
Supplement: SUPPLEMENTARY DATA [file supp_43_2_732__index.html]

Inter-individual variability contrasts with regional homogeneity in the human brain DNA methylome — Inter-individual variability contrasts with regional homogeneity in the human brain DNA methylome — SUPPLEMENTARY DATA 

# Inter-individual variability contrasts with regional homogeneity in the human brain DNA methylome

## SUPPLEMENTARY DATA

**Files in this Data Supplement:**

- SUPPLEMENTARY DATA
- SUPPLEMENTARY DATA
