# Supplementary material for: Inter-individual variability contrasts with regional homogeneity in the human brain DNA methylome
Source: Nucleic Acids Res. 2015 Jan 8;43(2):732–44. doi: 10.1093/nar/gku1305 (PMC4333374; doi:10.1093/nar/gku1305)
Supplement: SUPPLEMENTARY DATA [file supp_gku1305_Supplementary-Figures.pdf]

Supplementary Figures

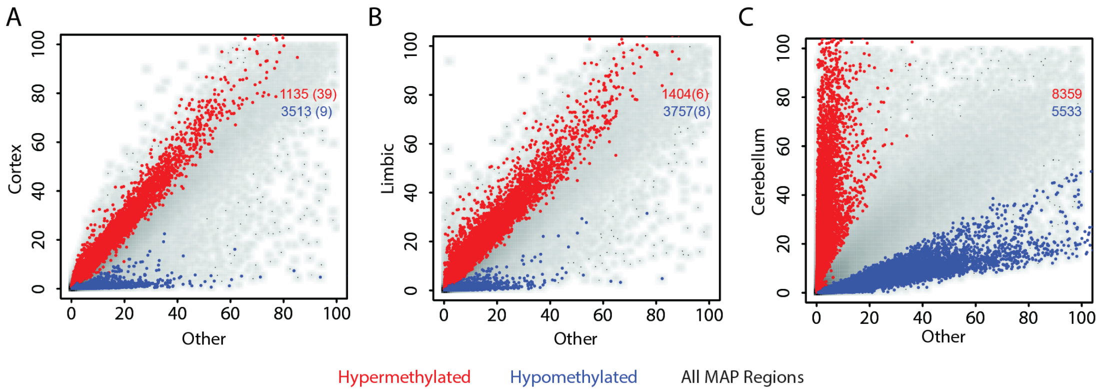

Supplementary Figure 1. Scatter plots indicating that the cerebellum methylome is highly distinct from both cortical and limbic brain compartments. The number of hypermethylated (red) and hypomethylated (blue) DMRs are indicated for each comparison. In parenthesis are the numbers of DMRs which are not attributed to the differences arising specifically from Cerebellum DMRs (plots are presented as for Figure 2A).

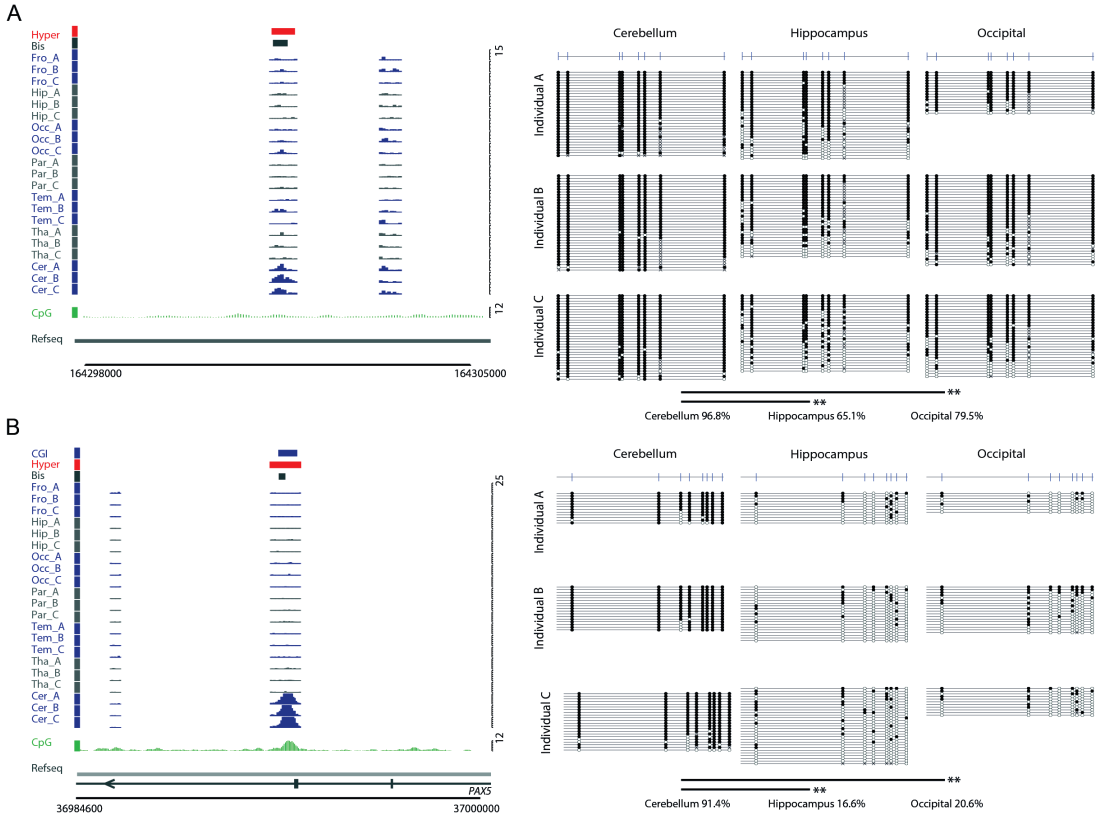

Supplementary Figure 2. Bisulfite validation of cerebellum DMRs. (A) The left panel depicts the MAP-seq profiles for each brain region (dark blue and grey) at an intergenic locus (chr6 164297500–164365143; hg18 genome build). The right panel shows the bisulfite sequencing result confirming that cerebellum is more heavily methylated relative to occipital and hippocampal samples in all three individuals as predicted by the MAP-seq analysis. B) The left panel depicts the MAP-seq profiles for each brain region (dark blue and grey) at an intragenic portion of the *Pax5* locus (chr9 36984000–37000000; hg18 genome build). The right panel shows the bisulfite sequencing result, which confirms that cerebellum is more heavily methylated relative to occipital and hippocampal samples in all three individuals as predicted by the MAP-seq analysis. The colouring and layout is presented as for Figure 2B.

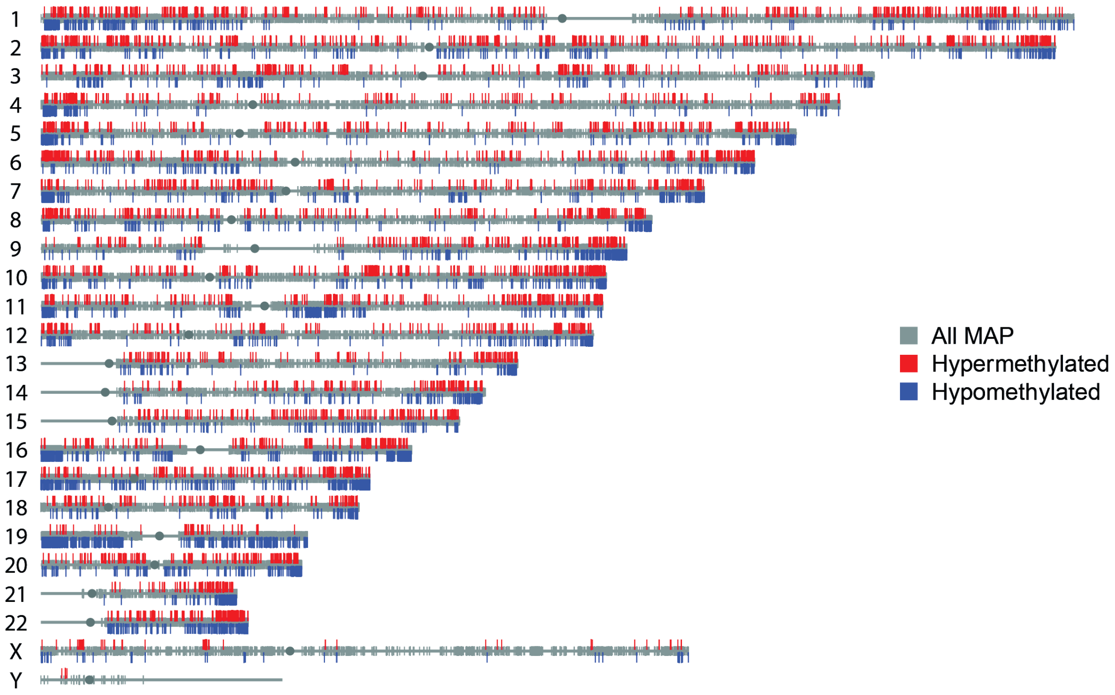

Supplementary Figure 3. Karyotype image of cerebellum-specific hyper- or hypo-methylated DMRs (red and blue respectively) vs. all MAP-regions (grey) show that they have distinct genomic distributions.

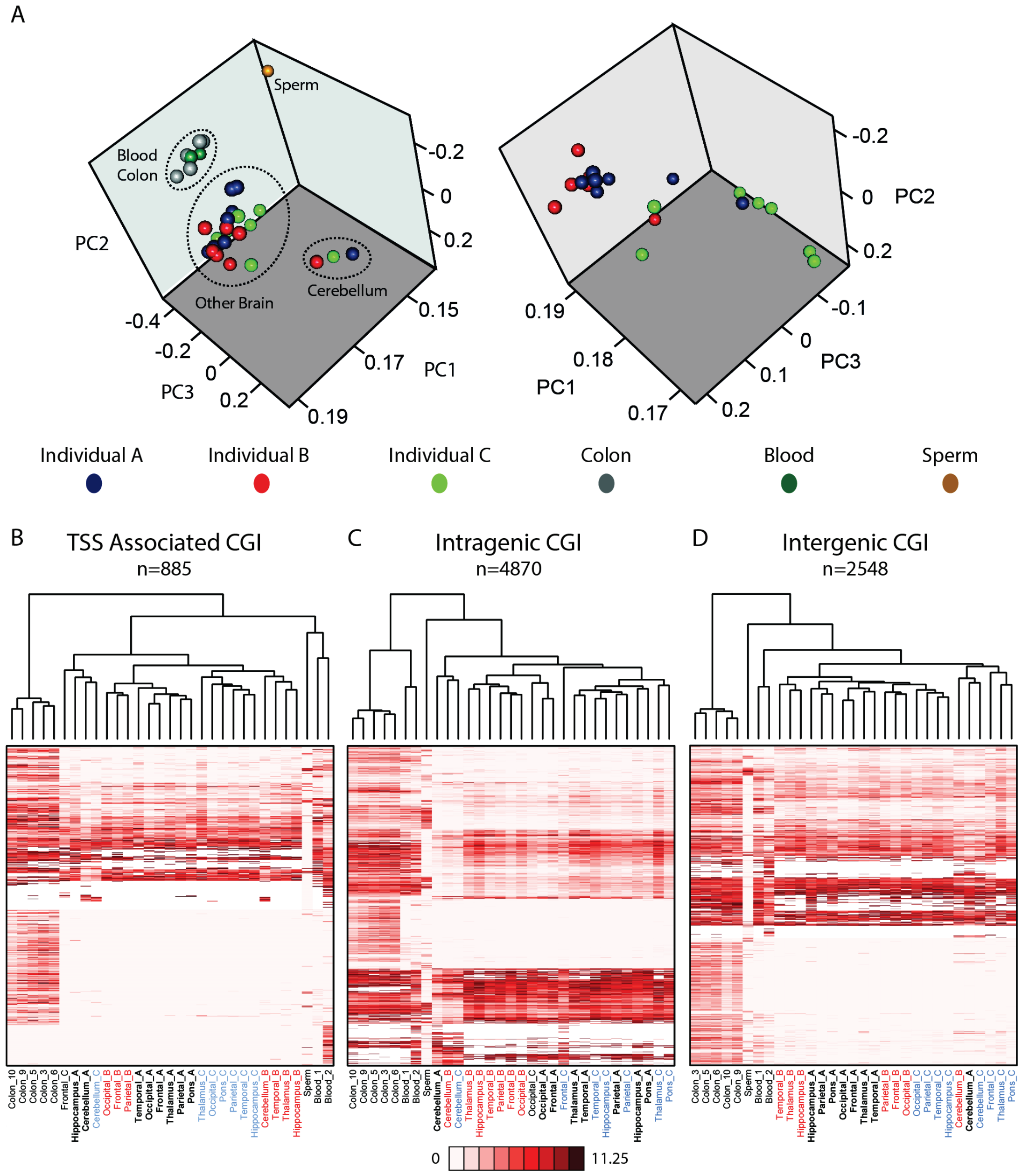

Supplementary Figure 4. High level concordance between the methylomes of different brain regions from the same individual. (A) Plots depicting the first three principle components which define the methylation patterns in all 31 samples presented in Figure 4 (left panel) and a zoomed and reoriented view of the 'Other Brain' region group (excluding cerebellum; right panel). (B-D) Heatmaps depicting the result of hierarchical clustering identifies a high concordance between different brain regions from the same individual at different categories of CGIs. The heatmaps show the average read depth for all TSS (B), intragenic (C) and intergenic (D) CGI associated MAP-regions. The number of MAP regions (n) in each analysis is shown. Layout is presented as for Figure 4.

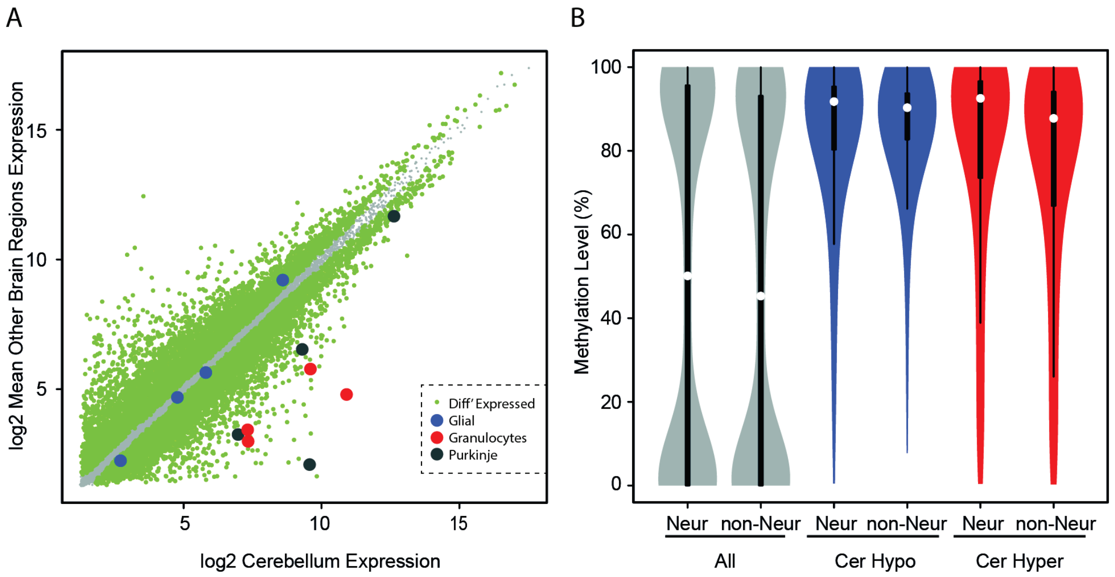

Supplementary Figure 5. Neuronal and not glia composition distinguish cerebellum from other brain regions. (A) Cerebellum gene expression versus the average expression for other brain regions indicates that markers of glia (blue) are not enriched in either dataset whilst neuronal markers (purkinje cells, grey; granule cells, red) are enriched in the cerebellum. Genes showing significantly differential gene expression are indicated in green ($p < 0.01$). (B) Violin plots depicting averaged eRRBS data for neuronal and non-neuronal cell fractions for all CpGs (grey), or for hypo- and hyper-methylated cerebellum DMRs specifically (blue and red respectively).

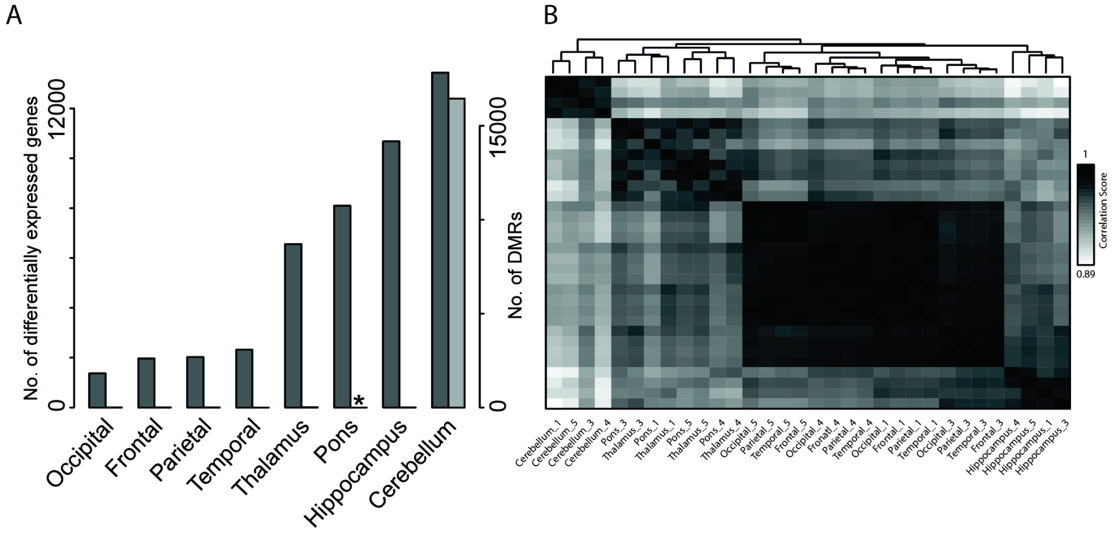

Supplementary Figure 6. The primary variable that dictates gene expression levels is individual of origin rather than brain region. (A) Bar plots depicting the number of differentially expressed genes (dark grey bars) and DMRs (light grey bars) for each brain region shows that major differences in gene expression occur despite a relatively constant epigenome. *DMR information for pons is not presented due to insufficient samples to perform the analysis. (B) The correlation heatmap shows that gene expression profiles in different cerebella are highly similar, but that other brain regions cluster preferentially by individual (n=4). The dendrogram represents the result of hierarchical clustering on which the heatmap sample order is based. The scale indicates Pearson correlation score (R).

A

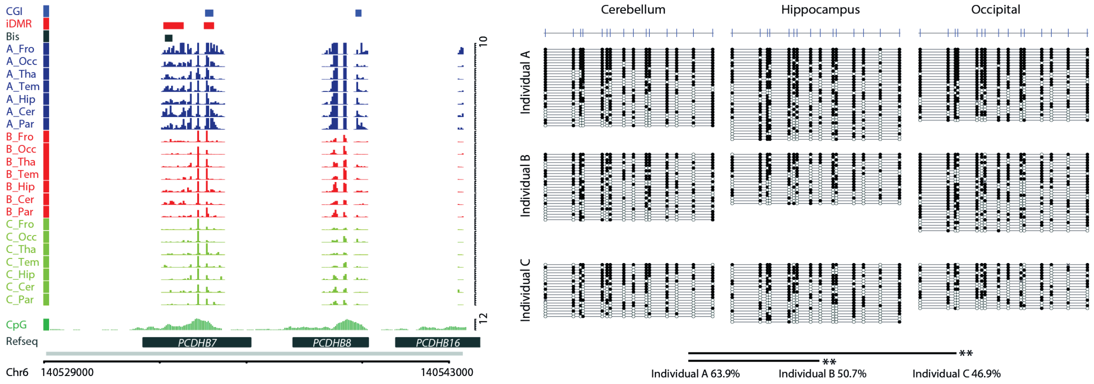

Supplementary Figure 7. Confirmation of an inDMR overlapping the *PCDHB7* locus by bisulfite genomic sequencing. (A) The left panel depicts the MAP-seq profiles for each individual (Individual A, darkblue; Individual B, red; Individual C orange) at the *PCDHB7* locus (chr5 140528475–140543475; hg18 genome build). The right panel shows the bisulfite sequencing result confirming that the methylation level is highest in individual A and lowest in C as predicted by the MAP-seq analysis. The layout is presented as for Figure 2B.

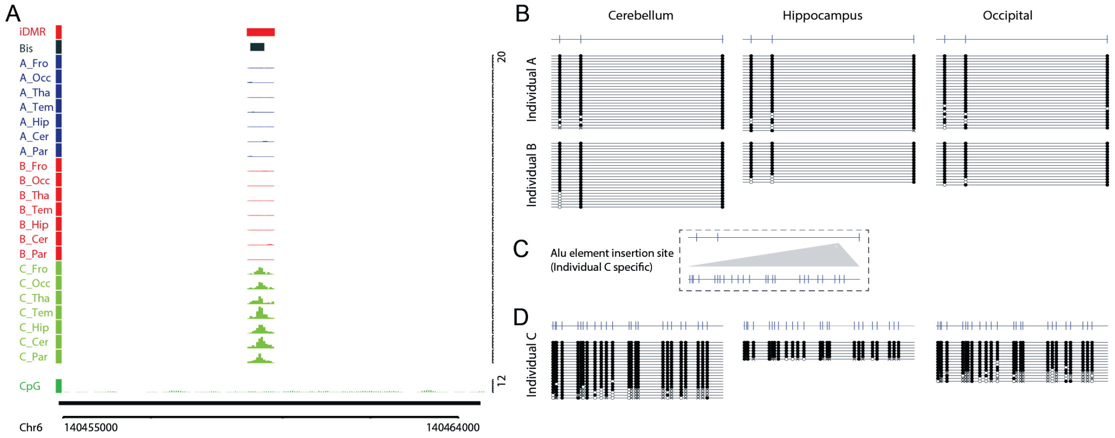

Supplementary Figure 8. Individual specific insertion of a methylated Alu element. (A) Depicts the MAP-seq profiles for each individual (A, darkblue; B, red; C orange) at an intergenic locus on chromosome 6 (chr6 140454500–140464500; hg18 genome build). (B) The DMR was shown to be methylated for individuals A and B but could not be determined for individual C. (C) Schematic representation of the insertion site and CpG map (denoted by vertical blue strokes) of a heavily methylated Alu element (shown in D). (A, B and D) are presented as for Figure 2B.

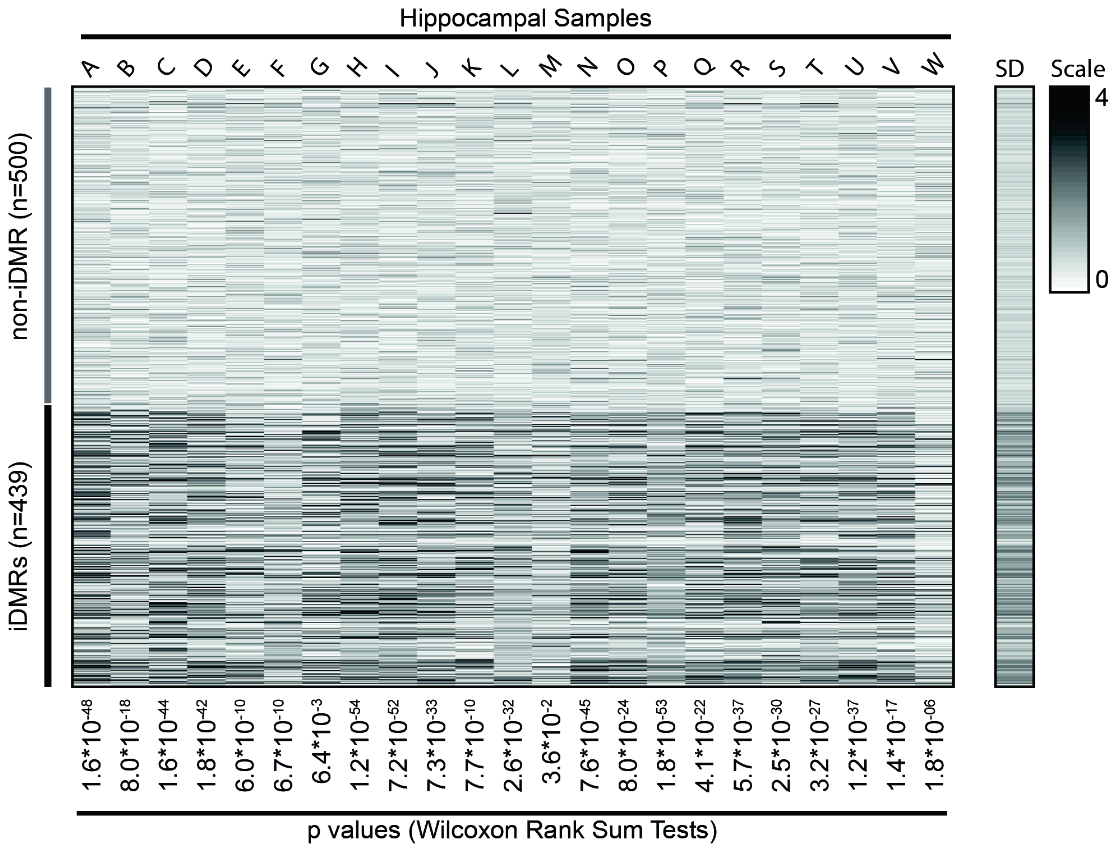

Supplementary Figure 9. InDMRs have hyper-variable MAP-seq signal in an extended panel of twenty-three hippocampal samples. The heatmap shows the log2 ratio for the MAP-seq signal form each individual vs. the mean from all individuals (the scale bar represents log2 ratios between the individual and the mean MAP-seq score irrespective of whether the differential is a relative loss or gain of methylation). The heatmap is separated into non-inDMR (upper) and inDMR (lower) associated MAP-regions. Standard deviations (SDs) for each MAP-region are indicated on the right of the panel. For each individual, the inDMRs have a significantly higher SD score than all MAP-regions that lack inDMRs (as determined from analysis of individuals A-C; p values are indicated and represent Wilcoxen Rank sum test statistics).

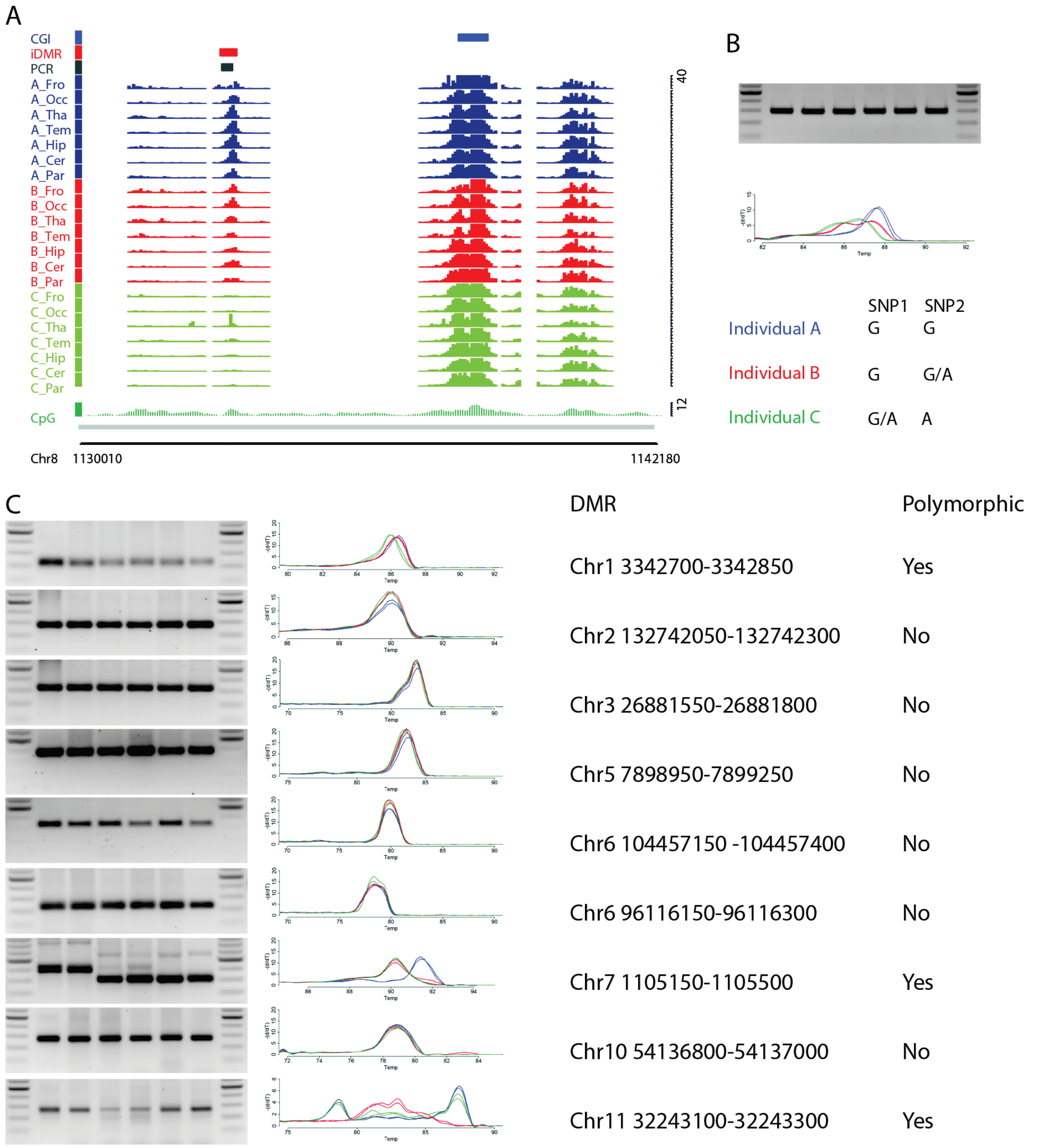

Supplementary Figure 10. DNA sequence polymorphisms can manifest as epigenetic variability. (A) Depicts the MAP-seq profiles for each individual (A, dark blue; B, red; C, orange) at a locus on chromosome 8 (chr8 1129500–1142175; hg18 genome build) containing a putative inDMR which was invalidated by bisulfite genomic sequencing (data not shown). (B) Quantitative PCR amplification of the inDMR generated products of uniform length but with distinct amplicon melt profiles (upper panel). Sanger sequencing confirmed that differential melt properties were due to differential representation of 2 single nucleotide polymorphisms within the inDMR (lower panel). (C) QPCR melt curve analysis of a further 9 putative inDMRs identified 3 as being polymorphic with the potential to confound accurate sequence mapping.
